# Orchid Biochemistry

**DOI:** 10.3390/ijms21072338

**Published:** 2020-03-27

**Authors:** Jen-Tsung Chen

**Affiliations:** Department of Life Sciences, National University of Kaohsiung, Kaohsiung 811, Taiwan; jentsung@nuk.edu.tw

Orchids belong to Orchidaceae which is one of the largest families in flowering plants. This family comprises over twenty thousand members, and many of them are fascinating with attractive flowers that sell in the markets with increasing demand around the world. What often receives less attention is the fact that some orchids are edible or scented, and more than this, many species have long been used in preparations in traditional medicine. The Special Issue “Orchid Biochemistry” collected original research and review articles that explore molecular aspects and insights into pigment formation, floral scent and pollination, bioactive compounds, plant-microbial interaction as well as biotechnology in orchid species.

## 1. Pigment Formation

Orchid populations have always been good materials for revealing the secrets of plant evolution. Zhang et al. studied species evolution using comparative transcriptomics in the *P. limprichtii* population that has a wide range of floral color varieties [1]. They proposed that the distribution pattern of different color morphs may be considered as a reproductive strategy that plays an important role in maintaining the population size. In this study, a molecular mechanism of color variation was proposed in which a crucial gene *PlFLS* interacts with a putative MBW protein complex (MYB, bHLH, and WDR), which may sever as a repressor of anthocyanin accumulation. 

Flower spot patterning could affect the ornamental value of some orchids and may play a significant role in the interaction with pollinators. Zhao et al. used a transcriptome analysis in the anthocyanin biosynthetic pathways of *Phalaenopsis* “Panda” to identify differentially expressed genes (DEGs) [2]. They further confirmed that some candidate structure genes among the DEGs expressed in significantly higher levels in spot tissues using qPCR analysis. Eventually, differentially expressed miRNAs (DEMs) were analyzed and 40 DEMs target transcription factor genes were found to express in significantly different levels in the spot sepal. According to the results, they proposed a microRNA-suppressing model for explaining the regulation in flower spot formation.

A comparative metabolomic study was made by Gao et al. and aims to reveal the regulation of flavonoid biosynthesis that contribute to leaf color formation in a foliage orchid, *Cymbidium sinense* “Red Sun” [3]. They identified 196 flavonoid-related metabolites using a UPLC-MS/MS-based method and revealed that the trends of leaf color changing from red to yellow and eventually to green, were mainly contributed by down-regulated anthocyanin biosynthetic enzymes.

## 2. Bioactive Compounds

*Dendrobium* orchids possess a number of bioactive compounds that have been used as traditional Chinese medicine for thousands of years. In recent years, a number of *Dendrobium* transcriptomes have been announced, and it opens a way to predict gene functions via in silico analysis. Zhang et al. performed a comparative analysis of *PLP_deC* genes in *D. officinale*, and the results showed that they may be involved in the responses of abiotic stress and consequently affect the biosynthesis of secondary metabolites [4]. Yuan et al. used weighted gene co-expression network analysis to predict crucial gene modules that may be involved in the regulation and biosynthesis of active compounds in *Dendrobium* orchids [5].

*Bletilla striata* (Thunb.) Reichb.f is an important traditional Chinese herb with multi-bioactivities. A dihydrophenanthrene compound, coelonin, was isolated and identified by Jiang et al. [6]. This compound mainly has anti-inflammatory activity, and the negative regulator phosphatase and tensin homologue on chromosome ten (PTEN) may play a crucial role in inhibiting macrophage proliferation and inflammatory factor secretion when treated silicosis.

## 3. Flower Scent

Bohman et al. studied the mechanism of pollination in European *Ophrys* orchids, and they identified two new pollinator attractants, including (*Z*)-8-Heptadecene and *n*-pentadecane, and gained insights into the biosynthesis of semiochemicals [7]. Ramya et al. contributed a review to summarize the advances of volatile organic compounds in orchids mainly focusing on their gene expression patterns in different tissues and developmental stages of *Cymbidium* orchids as well as their key role in pollination ecology [8]. They proposed a molecular breeding strategy through the manipulation of floral traits to improve the quality of orchids in the future.

## 4. Plant-Microbial Interaction

Orchids commonly have a symbiotic relationship with mycorrhizal fungi that benefit seed germination, seedling growth, and development. Sarsaiya et al. identified five species of myco-endophytes in *Dendrobium* orchids, and subsequent in vitro testing showed that they could affect seedling growth, especially at the stage of protocorms [9]. Zhang et al. profiled metabolome and transcriptome in the symbiosis between a mycorrhizal fungus, *Ceratobasidium* sp. AR2, and a medicinal orchid, *Anoectochilus roxburghii* [10]. They concluded that *C.* sp. AR2 could induce differential expressions, particularly in flavonoid biosynthetic genes and accomplished an increase in the accumulation of some flavonoids. They proposed that *C.* sp. AR2 has a high potential to enhance the quality of *A. roxburghii*.

## 5. Biotechnology

Orchids have a unique structure that induces from explants in vitro, namely protocorm-like bodies (PLBs) that resemble or equate to somatic embryos. Cardoso et al. provided an overview of PLBs in aspects of biotechnology and molecular biology [11]. Commonly, orchid PLBs are adequate materials for studying developmental biology and breeding techniques. In this review, they suggested that techniques using induction, proliferation, and regeneration of PLBs could be applied in the commercial mass propagation of orchids in the future.

## 6. Conclusions and Perspectives

Overall, this Special Issue collected recent advances in orchid biochemistry, including original articles and reviews. It provides in-depth insights into the biology of pigment and flower scent formation, bioactive compounds, and plant-microbial interaction as well as the biotechnology of PLBs. With the rapid progress of high-throughput technologies and integrative omics, scientists may have more opportunities to reveal the secret of orchid biology in the future.
